# Case Report: Atrial baffles of pulmonary and systemic veins for the anatomic and physiologic repair of left atrial isomerism heterotaxy—pre- and post-operative three-dimensional reconstructions of two mirror-image pediatric hearts

**DOI:** 10.3389/fcvm.2025.1529485

**Published:** 2025-03-20

**Authors:** Gregory Perens, Michael Silberbach, Jeffrey Frazer

**Affiliations:** ^1^ Department of Pediatrics, UCLA, Los Angeles, CA, United States; ^2^ Doernbecher Children's Hospital, Oregon Health & Science University, Portland, OR, United States; ^3^ Department of Pediatrics, Santa Barbara Cottage Hospital, Santa Barbara, CA, United States

**Keywords:** heterotaxy syndrome, imaging—computed tomography, congenital heart abnormalities, three-dimensional, surgery

## Abstract

Intra-atrial baffles of pulmonary and systemic venous flows are relatively rarely used but are important surgical procedures for late-presenting d-transposition of the great arteries (D-TGA) or heterotaxy with anomalous venous connections. Atrial baffle surgery is employed less frequently today due to the advent of the arterial switch procedure. Two children presented with mirror-image venous connections in polysplenic heterotaxy and underwent intra-atrial baffle biventricular repairs. Both cases had ipsilateral pulmonary venous connections, interrupted inferior vena cava with azygous continuation, and concordant ventriculo-arterial connections. Furthermore, a single superior vena cava receiving the azygous and hepatic veins connected to the atrium contralateral to the sub-pulmonary right ventricle was present in both cases. Volumetric cardiac imaging using computed tomography and magnetic resonance imaging allowed for the creation of three-dimensional (3D) model reconstructions for pre-operative surgical planning and postoperative assessment of the atrial baffle pathways. The 3D model reconstructions presented here provide improved visualization and understanding of complex surgical atrial baffles.

## Introduction

Intra-atrial baffles of pulmonary and systemic venous flows are relatively rarely used but are important surgical procedures for late-presenting d-transposition of the great arteries (D-TGA) or heterotaxy with anomalous venous connections. Two children presented with mirror-image venous connections in polysplenic heterotaxy with cardiac segments {A,L,I} and {A,D,S} and underwent intra-atrial baffle biventricular repairs. Both cases had ipsilateral pulmonary venous connections, interrupted inferior vena cava (IVC) with azygous continuation, and concordant ventriculo-arterial connections. Furthermore, a single superior vena cava (SVC) receiving the azygous and hepatic veins connected to the atrium contralateral to the sub-pulmonary right ventricle was present in both cases. Volumetric cardiac imaging using computed tomography (CT) and magnetic resonance imaging (MRI) allowed for the creation of three-dimensional (3D) model reconstructions for pre-operative surgical planning and postoperative assessment of the atrial baffle pathways. The 3D model reconstructions presented here provide improved visualization and understanding of complex surgical atrial baffles.

## Background

The first successful surgical repair of D-TGA used the atrial baffle (1). This procedure uses the patient's own cardiac tissue to baffle the normally connected systemic veins to the sub-pulmonary left ventricle and the pulmonary veins to the morphologic sub-aortic left ventricle. The procedure was modified by Mustard using external materials. Once the arterial switch, initially performed by Jatene et al., became routine (2), the atrial switch was used much less frequently. However, the atrial switch for D-TGA remains important for D-TGA cases presenting later in life and for those who are not candidates for arterial switch (3, 4). Furthermore, the anomalous venous connections in heterotaxy may require intra-atrial baffling for either a physiologic (morphologic left ventricle) or a full anatomic (sub-pulmonary morphologic right ventricle) repair. Biventricular repair for left isomerism was reported as early as 1995 (5).

An atrial baffle for the venous anatomy in hearts with heterotaxy is not necessarily the same as that in hearts with normal venous anatomy with D-TGA. Anomalous venous connections are common in both asplenia and polysplenia heterotaxy. While specific venous anatomy is common to one or the other, such as interrupted IVC and ipsilateral pulmonary veins in polysplenia, a variety of other arrangements of the superior vena cava, hepatic veins, and total anomalous pulmonary venous drainage are possible. A subset of heterotaxy cases can result in a biventricular repair (6–8). When two adequate ventricles are present, the venous anatomy must be septated to create either a physiologic repair with a morphologic sub-aortic right ventricle or a full anatomic and physiologic repair with a concordant ventriculo-arterial connection.

3D model reconstruction and printing from cardiac MRI and CT have been described for pre-surgical planning to determine the feasibility of biventricular repair, most notably in hearts with a double-outlet right ventricle. The use of 3D printing in surgical planning for complex anomalous venous connections is becoming more commonly reported (8–10), and they include cases requiring various types of atrial baffles (8, 11). While the clinical outcomes of cases using 3D coronary heart disease (CHD) models have been reported, little has been reported on the 3D representations of the post-surgical anatomy.

Herein, we present 3D renderings of the pre-and post-surgical anatomy of two cases of heterotaxy with mirror-image abnormal venous anatomy who underwent atrial baffle repair.

## Methods

A retrospective review of two pediatric cases with similar cardiac anatomy was performed. Approval for the case series was obtained from the Institutional Review Board of the University of California, Los Angeles (UCLA). Patient charts and imaging were reviewed for clinical data and included surgical operative notes and pre-and post-surgical advanced axial imaging with CT or MRI.

## Imaging

Four-dimensional cardiac magnetic resonance imaging using ferumoxytol, an iron-based contrast agent, on a 3.0 T scanner (Magneton TIM Trio, Siemens Healthineers, Malvern, PA, USA) was used for pre-operative imaging in Case 1 (12). Cardiac non-gated computed tomography was used for pre-operative imaging in Case 2 and electrocardiogram (ECG)-gated volumetric images were obtained on a Siemens Force dual-source CT scanner in both cases for postoperative imaging.

For 3D reconstruction of the images, segmentation of the contrast-enhanced blood pool was first performed using semi-automatic region-growing in the program ITK-SNAP (13). The 3D blood pool representation was then modified and hollowed using the free, online program Meshmixer (Autodesk, Inc., San Francisco, CA, USA). To create the post-operative models, the pulmonary and systemic venous blood pools were segmented separately. For Case 1, the region of the patch was separately segmented up to where it could be visualized intersecting with the atrial walls.

## Cases

**Figure 1:**
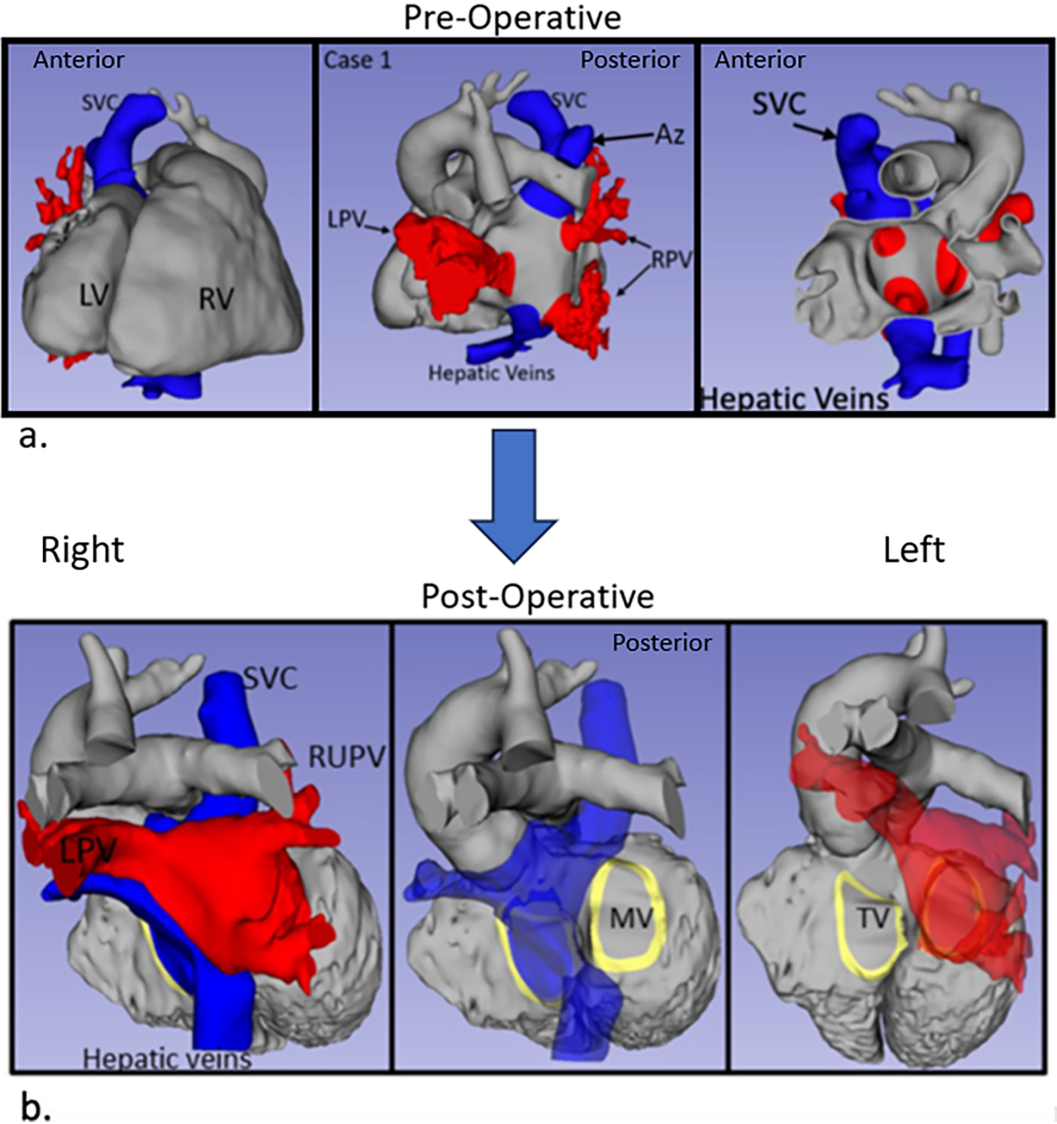
3D renderings derived from MRI in Case 1. **(a)** Pre-operative anatomy views shown from left to right: anterior, posterior, and anterior slice through the atria. **(b)** Post-operative anatomy. Systemic veins are colored blue and pulmonary veins are red. Az, azygous vein; LV, left ventricle; LPV, left pulmonary veins; RPV, right pulmonary veins; RV, right ventricle; SVC, superior vena cava.

**Figure 2:**
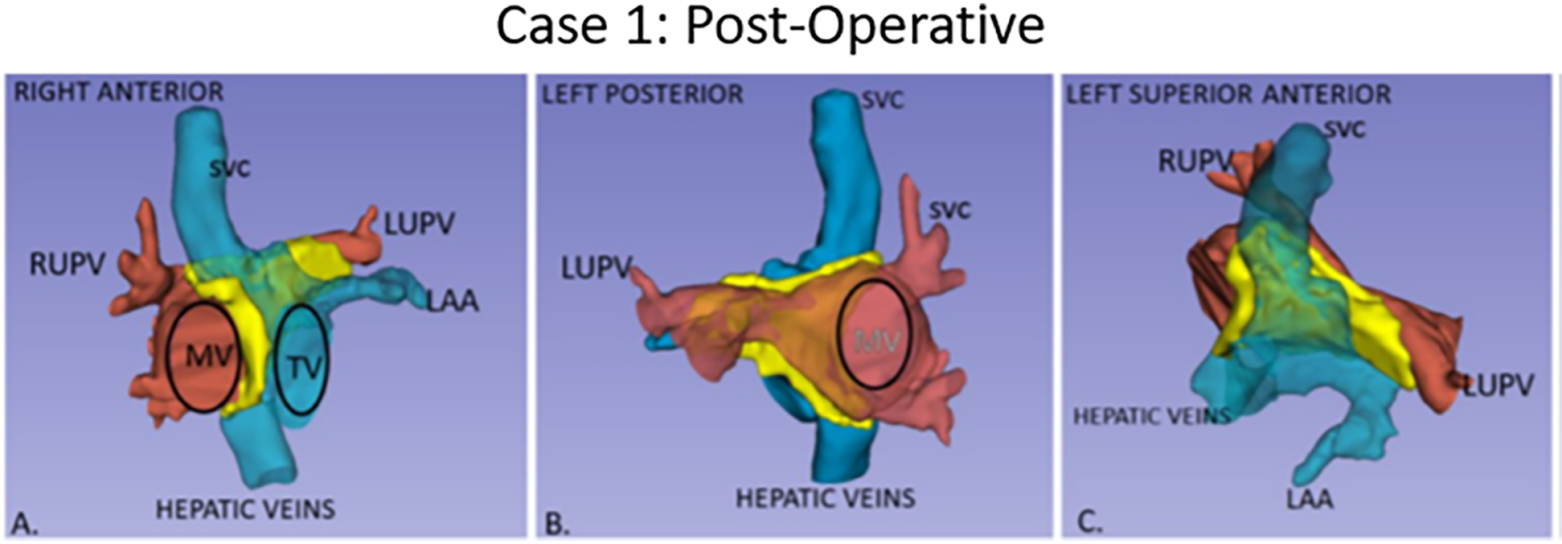
Post-operative anatomy in Case 1 with atrial baffle patch. Three-dimensional segmented blood pools of systemic (blue) and pulmonary (red) anatomy, with the surgical atrial baffle in yellow. **(A)** Right anterior view with partially transparent systemic veins. **(B)** Left posterior view with partially transparent pulmonary veins, and right-sided atrium. **(C)** Left anterior superior view.

Two pediatric patients, aged 3 years (Case 1) and 5 years (Case 2), presented for surgical repair of heterotaxy with abnormal venous anatomy. The pre-operative anatomy of each case is presented in Table 1 and Figures 1a, 2A. Both cases had dextroposition of the cardiac apex, venous anatomy consisting of an interrupted IVC with azygous continuation to a single SVC that drained into the atrium connected to the morphologic left ventricle, hepatic venous drainage on the same side as the SVC, ipsilateral pulmonary venous drainage, a common atrium, a cleft mitral valve, and ventriculo-arterial concordance. Both patients had baseline 02 saturations of 80%–90%. Each patient underwent cardiopulmonary bypass for atrial baffling to create a physiologic repair with the systemic morphologic left ventricle. Both had a repair of an isolated mitral valve cleft.

**Table 1:** Patient anatomy (Case 1, 3-year-old boy; Case 2, 5-year-old girl).

Cardiac anatomy component	Case 1	Case 2
Cardiac position/apex	Left/Right	Right/Right
Segments	{A,L,I}	{A,D,S}
Systemic veins	Interrupted IVC; azygous continuation to RSVC; right-sided hepatics	Interrupted IVC; azygous continuation to LSVC; left-sided hepatics
Pulmonary veins	Ipsilateral	Ipsilateral
Atrial defects	Common atrium	Common atrium
Ventricular-arterial connection	Concordant	Concordant
Additional cardiac diagnoses	Cleft mitral valve with redundant valve tissue in LVOT	Cleft mitral valve, bicuspid aortic valve; pulmonary valve stenosis; patent ductus arteriosus
Bronchi	Bilateral hyparterial	Bilateral hyparterial
Abdominal organs and lungs	Single spleen; transverse liver; intestinal malrotation	Polysplenia; intestinal malrotation; biliary obstruction; ciliary dyskinesia
Conduction system	Left atrial rhythm	Congenital complete heart block, bradycardia requiring pacemaker

RSVC, right superior vena cava; LSVC, left superior vena cava; LVOT, left ventricular outflow tract.

Case 1 had cardiac segments {A,L,I}. At surgery, an autologous pericardium was used. To avoid the conduction system in Case 1, care was taken to sew the patch to the left of its expected location in the region of the atrioventricular (AV) valves.

Post-operative transesophageal echocardiography (TEE) demonstrated no baffle obstruction, with a central venous pressure (CVP) of 8 mmHg. The patient was re-admitted 2 weeks post-operatively for left-sided chylothorax, which was treated with drainage and sclerotherapy. There were post-operative arrhythmias or heart blocks. Figure 1 shows the blood pool segmentations derived from the pre- and post-operative imaging. 3D segmentation of the atrial baffle patch separating the pulmonary and systemic venous flows is shown in Figure 2 and Supplementary Video S1.

**Figure 3:**
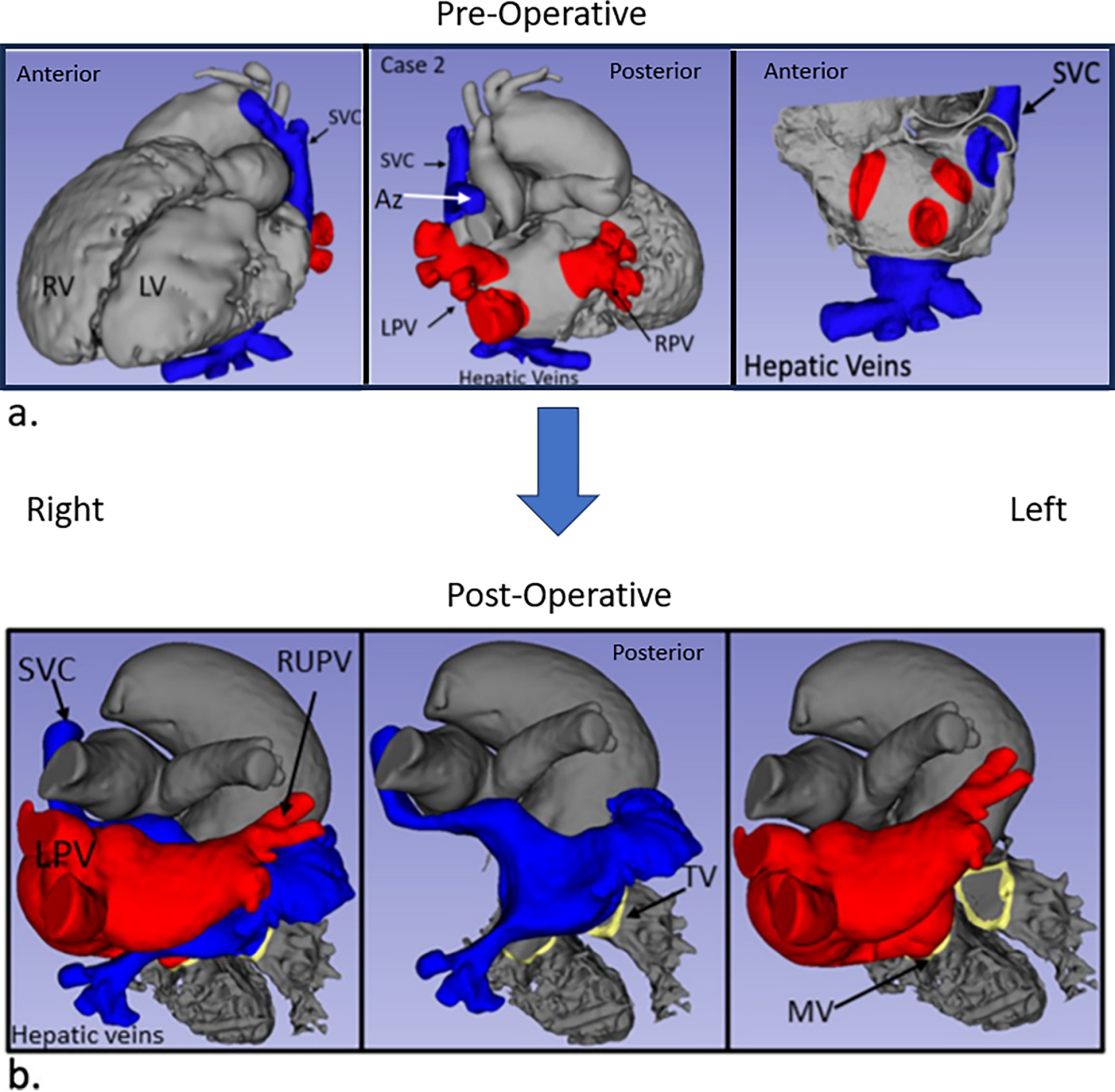
Case 2 imaging derived from CT. Pre-operative **(a)** and post-operative **(b)** images are shown with comparable views to Figures 1a,b.

Case 2 had segments {A,D,S} with surgery consisting of an atrial baffle (Figure 3), pulmonary valvuloplasty, and pacemaker generator change. Post-operatively, the mean echo Doppler gradient was 5 mmHg through the SVC baffle, and the patch and SVC were subsequently augmented, but the mean Doppler gradient remained at 5 mmHg. The central venous pressure in the SVC proximal to the atrium was 6 mmHg.

## Discussion

Three-dimensional reconstruction and 3D printing from MRI and CT have been demonstrated to assist in pre-procedural planning for complex CHD, often with the goal of a biventricular repair. Many forms of heterotaxy can ultimately undergo atrial and/or ventricular septation, resulting in a biventricular heart. The cases presented here had complex venous anatomy requiring intra-atrial baffles that differed from those classically used for D-TGA.

The two unique anatomic features that needed to be addressed were the increased flow in the SVC from the interrupted IVC and the ipsilateral pulmonary veins. The SVC baffle needed to be larger than a standard Senning or Mustard repair because it included flow from the interrupted IVC to the azygous vein. The 3D models were used to review the possible courses of the SVC flow to the contralateral AV valve. The SVC flow could be baffled inferiorly toward the hepatic vein entry or along the superior aspect of the atrium. In both cases, the SVC flow baffle was baffled superiorly, as shown in Figure 2. Despite surgical augmentation, Case 2 had a mean gradient of 5 mmHg through the SVC baffle, with an acceptable vena caval pressure of 6 mmHg. The flow gradient may have resulted from a relatively increased flow from both the SVC and interrupted IVC through this superior limb of the baffle and some lateral compression from a dilated left pulmonary artery. The CVP remained at 6 mmHg post-operatively and there was no facial or peripheral edema, so no further intervention was performed.

Second, the anomalous lower pulmonary veins that drained to the same side as the systemic veins connected to the atrium were very close to the inferiorly and anteriorly located hepatic veins in both hearts. The atrial baffle patch was extended inferiorly and anteriorly on the side of the systemic veins to separate the flows without obstruction (Figure 3).

Surgical repair of hearts with so-called isolated ventricular inversion, with atrioventricular discordance and ventriculo-arterial concordance, has been described using Senning- or Mustard-type repairs (14–16). Even more rarely, similar anatomies to those presented here, with left atrial isomerism, have been previously described (17, 18). However, imaging of the post-operative anatomy has been limited to 2D echocardiography and illustrations representing the surgical view.

While pre- and post-operative imaging with advanced CT and MRI is not required for surgical correction of left isomerism, axial and three-dimensional imaging does offer a complete view of the anatomy not otherwise visualized by a 2D or 3D echo. Experienced surgical groups have expounded on the usefulness of 3D modeling for congenital heart disease (9–11). The drawbacks of this approach are radiation with CT and, in younger patients, anesthesia.

## Limitations

There are limitations to 3D models created from CT and MRI. Most notably, valve leaflet tissue is usually not incorporated into the model unless it is very well imaged. In the cases presented here, the locations of the valve annuli were outlined based on their known locations on the source images. The study is limited in that it only reports a two-patient series. However, this anatomy is rare, and these cases are the only two with this venous anatomy that the authors have encountered at our institution with full-volume advanced imaging available for 3D reconstruction.

## Data Availability

The original contributions presented in the study are included in the article/Supplementary Material, further inquiries can be directed to the corresponding author.

## Supplementary material

Supplementary Video S1 Post-operative pulmonary and systemic venous flows and surgical atrial patch baffle.
